# Supplementary material for: Hybrid E/M Phenotype(s) and Stemness: A Mechanistic Connection Embedded in Network Topology
Source: J Clin Med. 2020 Dec 26;10(1):60. doi: 10.3390/jcm10010060 (PMC7794989; doi:10.3390/jcm10010060)
Supplement: Supplementary file 1 [file jcm-10-00060-s001.zip › jcm-1007161-supplement figures.docx]

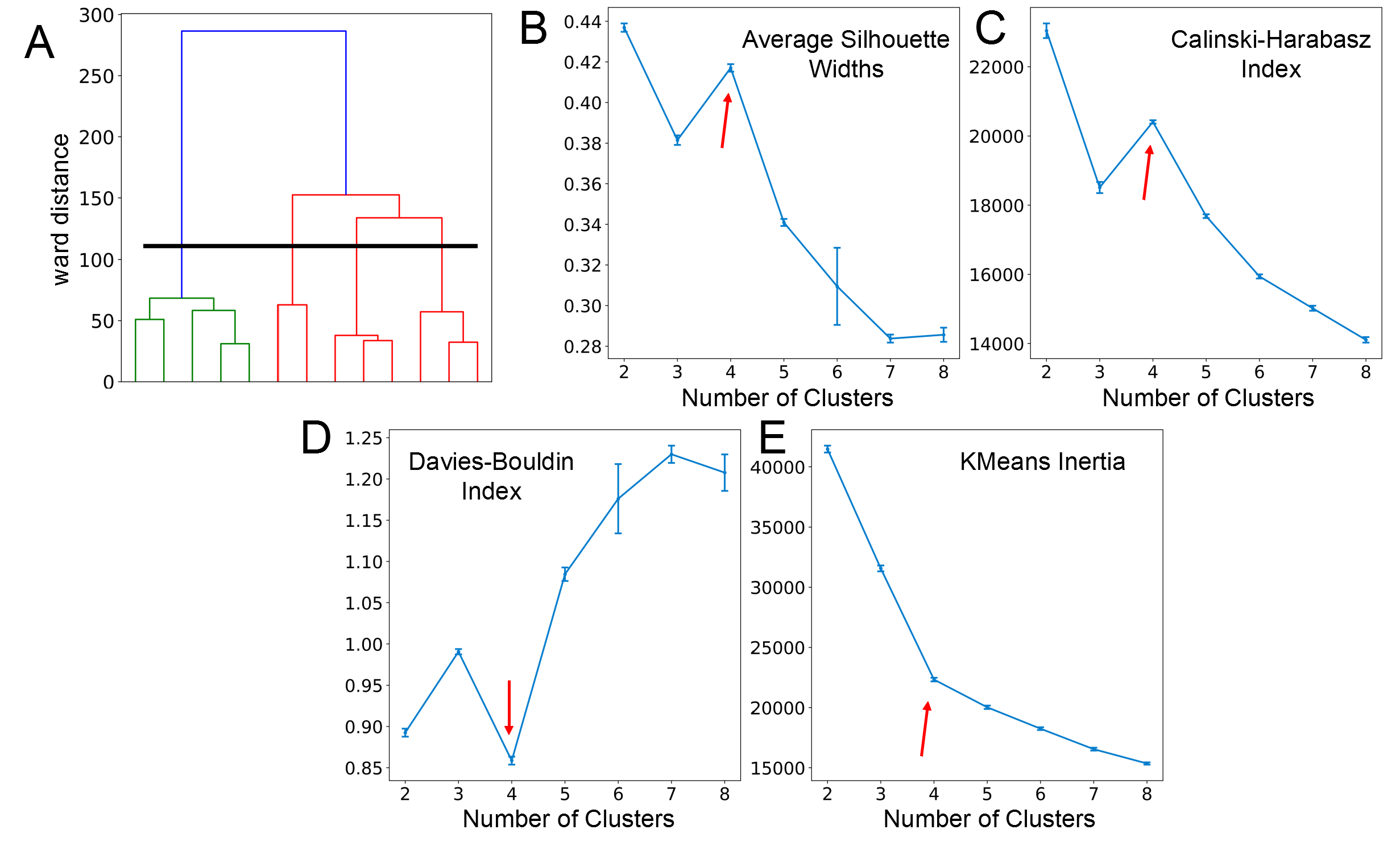
**Supplementary Figures**

***Figure S1: Optimal cluster number by varied cluster quality. A)*** *Dendrogram of a single replicate of the coupled EMP–stemness circuit computed using the Ward algorithm under agglomerative hierarchical clustering. The black horizontal line represents segregation of the dendrogram corresponding to a four-cluster solution.* ***B)*** *Average silhouette widths for the coupled EM–-stemness circuit at different numbers of clusters with a distinct peak (and a marked change in the slope) at cluster number = 4. The larger the average silhouette width, the more optimal is the clustering.* ***C)*** *Calinski–Harabasz index for the coupled EMP–stemness circuit at different numbers of clusters with a distinct peak (and a marked change in the slope) at cluster number = 4. The larger the index, the more optimal is the clustering.* ***D)*** *Davies–Bouldin index for the coupled EMP–stemness circuit at different numbers of clusters with a distinct dip (and a marked change in the slope) at cluster number = 4. The smaller the index, the more optimal is the clustering.* ***E)*** *K-means inertia (the objective of the K-means algorithm is to minimize this quantity iteratively) for the base circuit at different numbers of clusters with a distinct “elbow” (and a marked change in the slope) at cluster number = 4. All the values are averaged across n = 5 RACIPE replicates and the error bars represent the across-replicate standard deviations.*

***
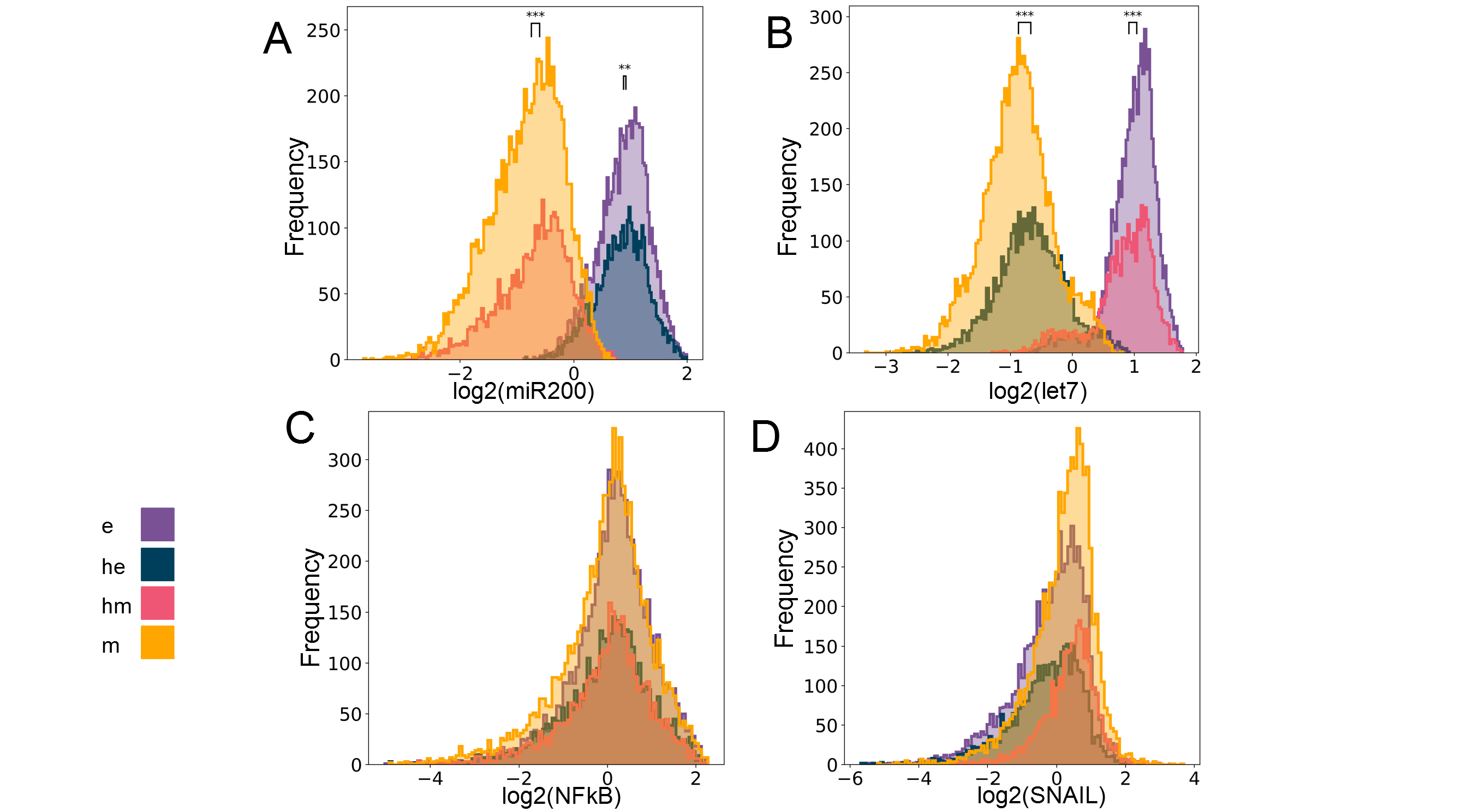
***

***Figure S2: Four E/M phenotypes enabled by the base circuit.***  ***A-D)*** *Expression histograms showing miR-200, let7, NF-kB, and SNAIL levels, respectively, in the four clusters—e, he, hm, and m. Significance bars for statistical testing (Mann–Whitney U test) added in B and C. Legend for the p-values: +: p > 0.01, *: 0.01 > p > 0.001, **: 0.001 > p > 0.0001, ***: p < 0.0001; for effect sizes, refer to* ***Table S7*** *in* ***Supplementary Section 2******.5****.*

*
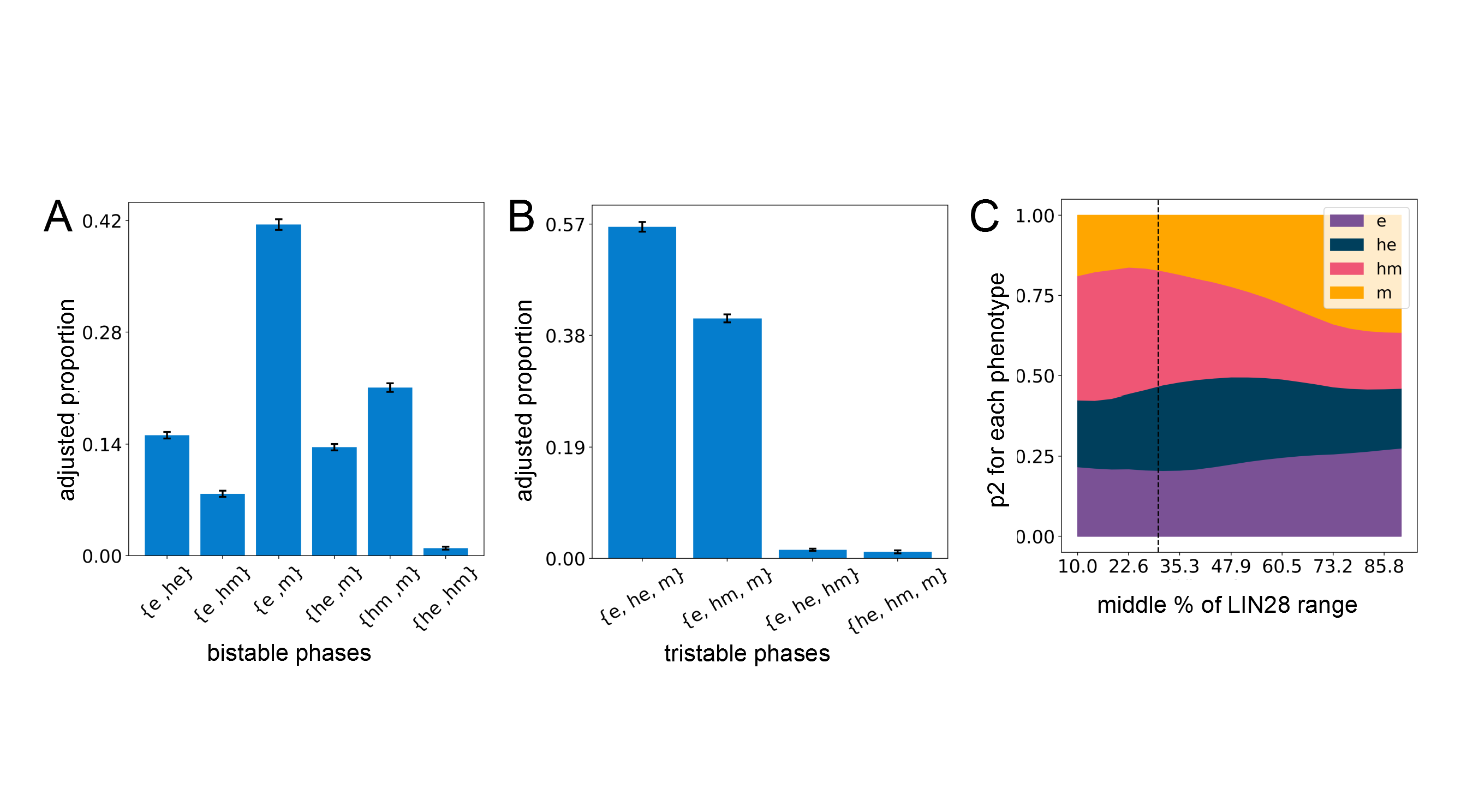
*

***Figure S3: Stemness window and the bistable/tristable phase distributions of the coupled EMP–stemness circuit. A)*** *Proportion of all bistable parameter sets in the coupled EMP–stemness (base) circuit belonging to different bistable phases, after removing the parameter sets with both solutions being assigned the same phenotype (see* ***Supplementary section 2.3****)****. B)*** *Same as A) but for tristable systems, after removing the systems with two or more solutions assigned the same phenotype****. C)*** *Sensitivity analysis by considering multiple sizes of the stemness windows centered at the median of the LIN28 distribution (averaged across n = 5 RACIPE replicates of the base circuit). The proportion of all high-stemness solutions belonging to each of the phenotypes (p2) is then plotted. The black vertical line corresponds to the 30% value taken as the stemness window and marks the approximate upper limit of the range with a relatively small change in the phenotype distribution upon altering the stemness window. A, B: The values are averaged across n = 5 RACIPE replicates and the error bars represent the across-replicate standard deviations.*


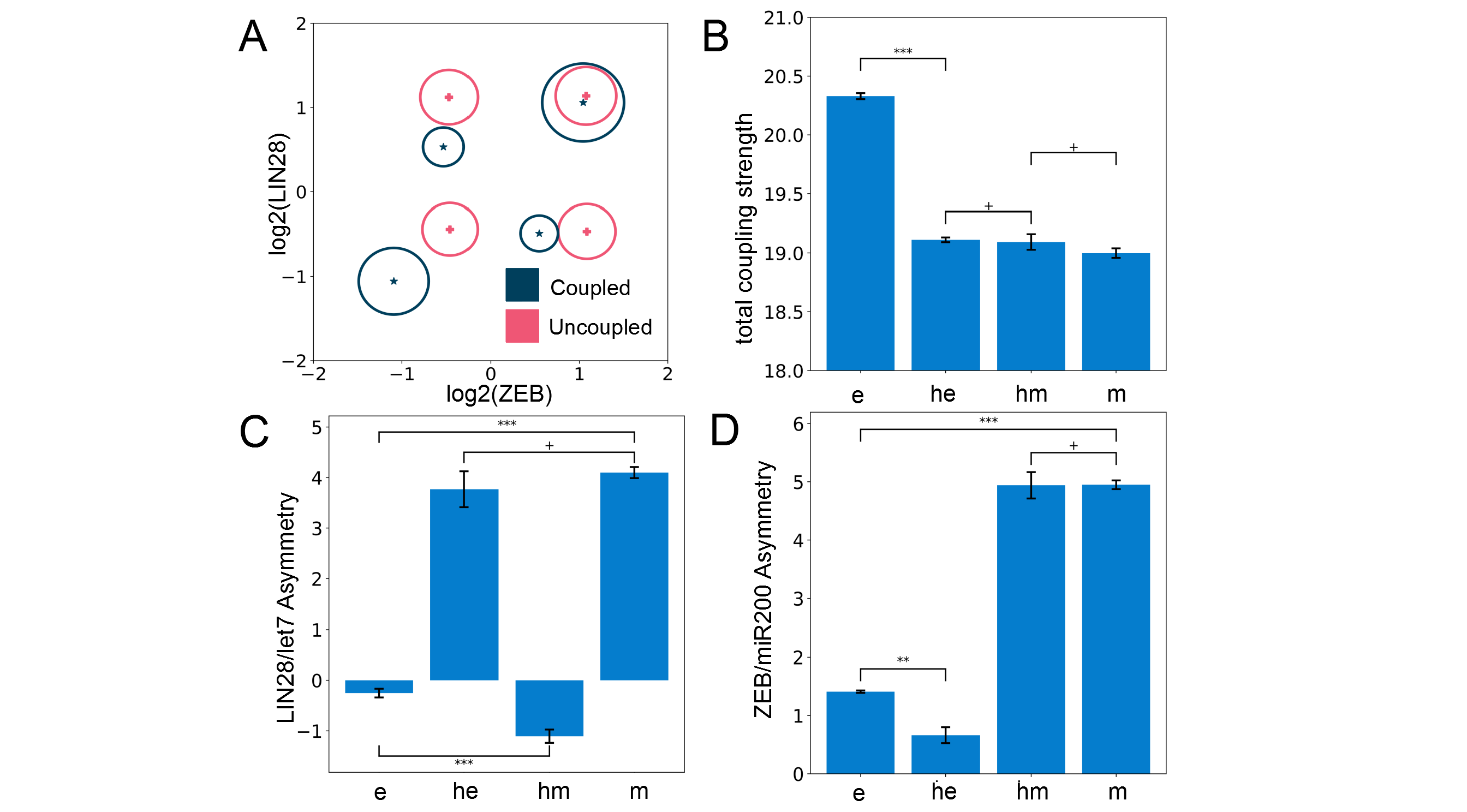
***Figure S4: Link strength analysis. A)*** *The cluster centers (median levels of ZEB and LIN28) in the ZEB–LIN28 plane for the coupled and the uncoupled EMP–stemness circuits. The radius of the circle around the center is proportional to the proportion of all solutions falling into the cluster. Both the uncoupled and the base circuit have been normalized with the coupled EMP–stemness circuit (see* ***Supplementary Section 2.2****)****. B)*** *The total coupling strength (see* ***Methods****) plotted for different phenotypes in the base circuit including only the parameter sets resulting in a monostable solution****. C)*** *Mean symmetry ratio (see* ***Methods****) of the LIN28/let-7 double negative feedback loop plotted for different phenotypes in the base circuit, including only the parameter sets resulting in a monostable solution****.*** *A higher ratio represents a stronger suppression of let7 by LIN28 in comparison to the reverse inhibition.* ***D)*** *Same as C) but for ZEB/miR-200 feedback loop. A higher ratio represents a stronger suppression of miR200 by ZEB in comparison to the reverse inhibition. B-D: The values are averaged across replicates and the error bars represent the across-replicate standard deviations. Welch’s t-test is used for significance. Legend for the p-values: +: p > 0.01, *: 0.01 > p > 0.001, **: 0.001 > p > 0.0001, ***: p < 0.0001; for effect sizes, refer to* ***Table S7*** *in* ***Supplementary Section 2******.5****.*

*
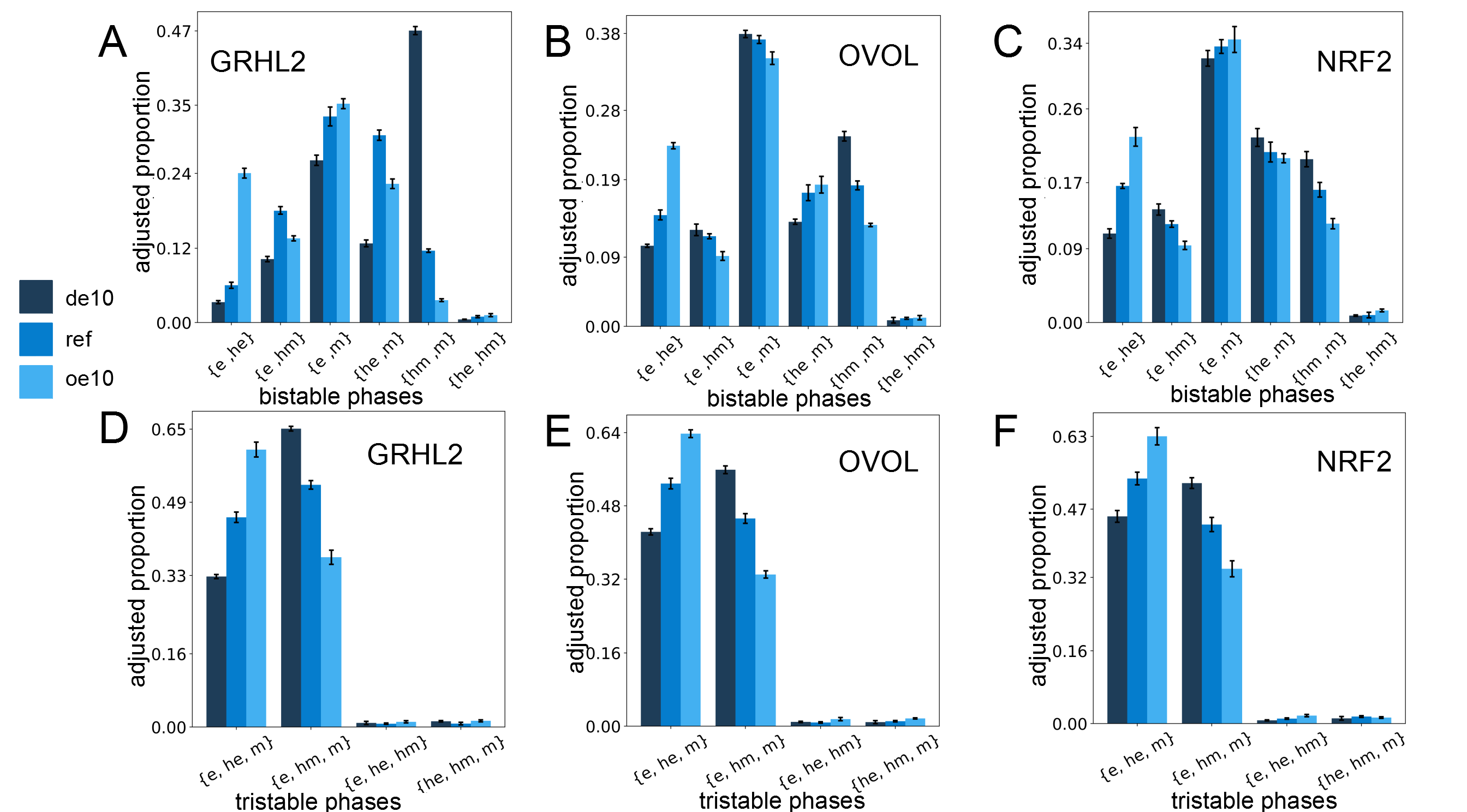
*

***Figure S5: Distribution of bistable and tristable systems upon incorporating PSFs with the coupled EMP–stemness circuit. A-C)*** *Frequency of different possible combinations of co-existing bistable states (i.e., phases) with varied expression of the PSFs for different circuits. Y-axis represents the proportion of all bistable systems belonging to different bistable phases after removing the systems with both solutions assigned the same phenotype (see* ***Supplementary Section 2.3****).* ***D-F)*** *Same as A-C but for tristable solutions after removing the systems with two or more solutions assigned the same phenotype (de10: 10-fold down-expression, ref: Reference unperturbed circuit, oe10: 10-fold overexpression). The values shown here are averaged across n = 5 RACIPE replicates and the error bars represent the across-replicate standard deviations.*


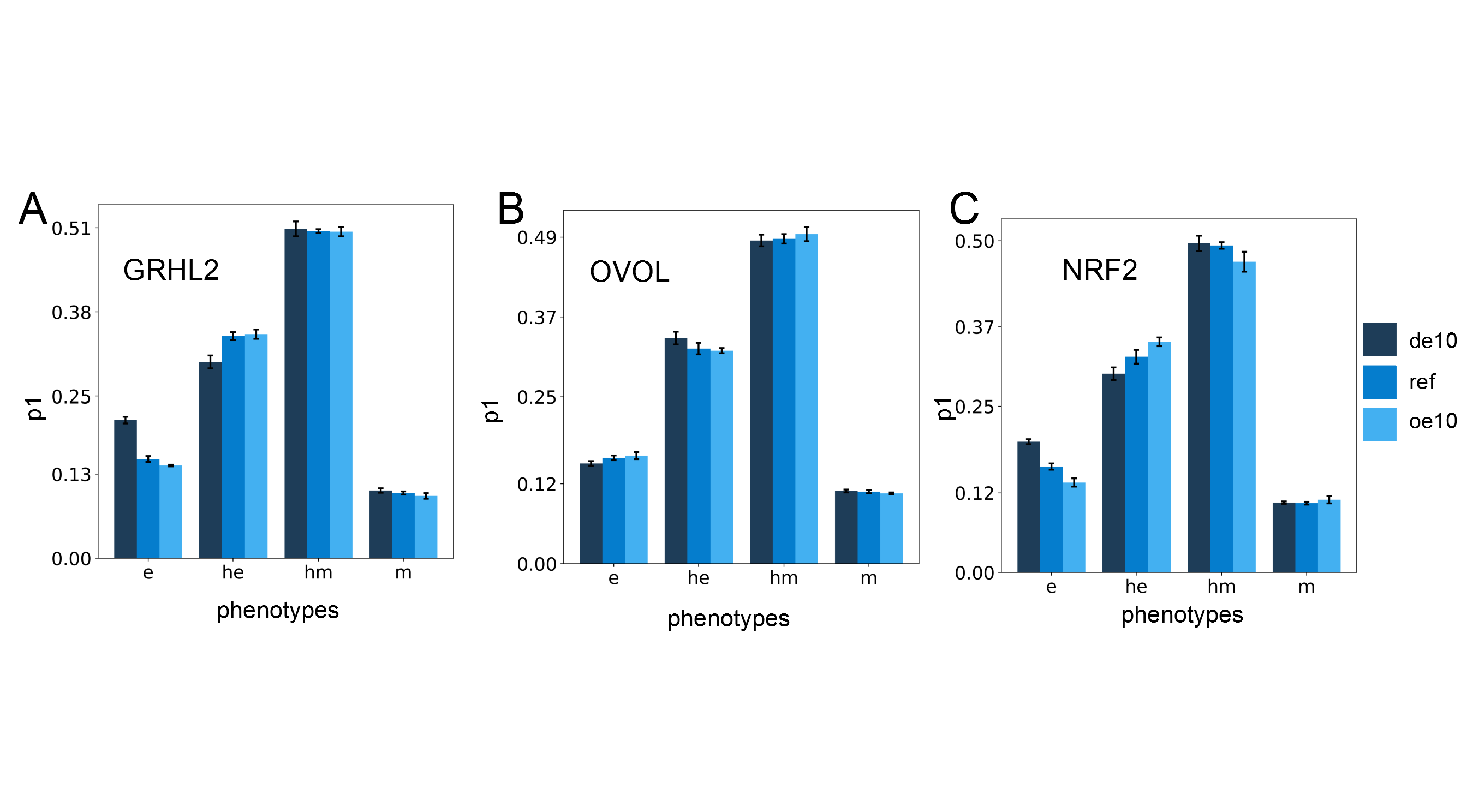
***Figure S6: Stemness probability (p1) changes with PSF expression. A-C)*** *Changes in the p1 probability for different PSF circuits at different expression levels of PSFs. See* ***Table S8*** *in* ***Supplementary Section 2.5*** *for the statistical testing for differences. (de10: 10-fold down-expression, ref: Reference unperturbed circuit, oe10: 10-fold overexpression).* *The values are averaged across n = 5 RACIPE replicates and the error bars represent the across-replicate standard deviations.*


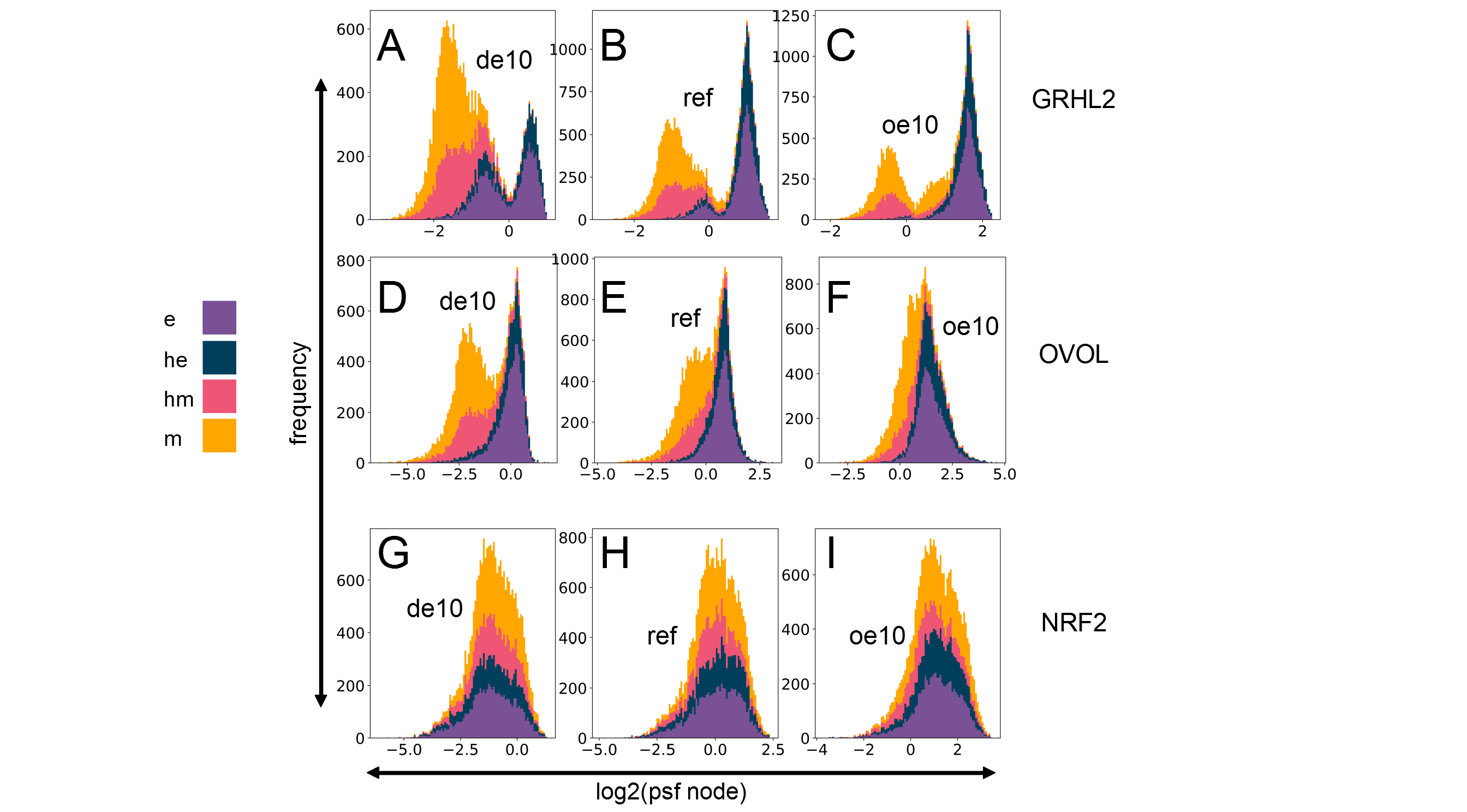
***Figure S7: PSF expression profiles at different expression levels. (A-C)*** *GRHL2 stacked histograms for a single RACIPE replicate (colored according to the phenotypes as shown in the legend) for different levels of expression of the GRHL2 node.* ***(D-F)*** *Same as A-C but for OVOL.* ***(G-I)*** *Same as A-C but for NRF2 (de10: 10-fold down-expression, ref: Reference unperturbed circuit, oe10: 10-fold overexpression).*


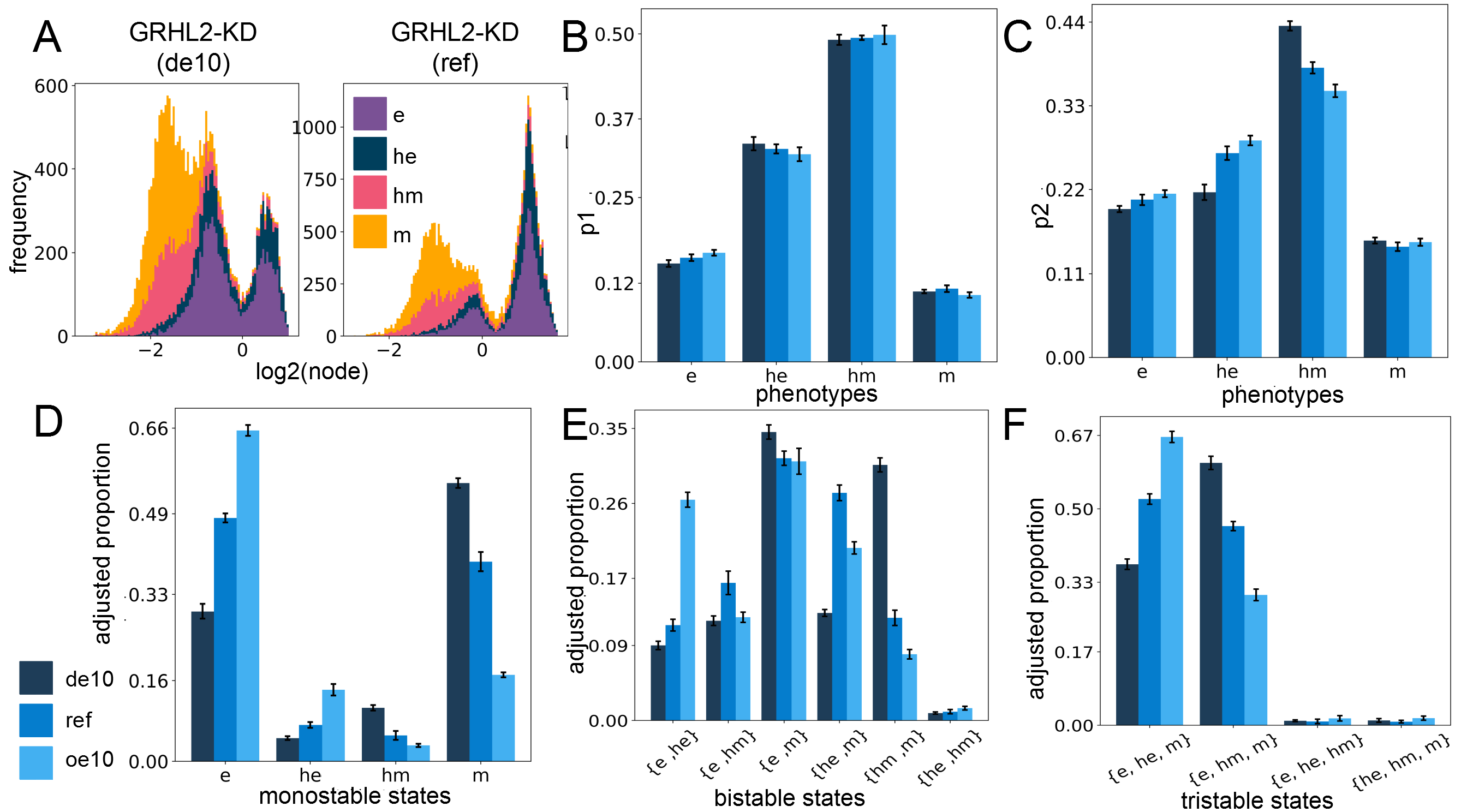
***Figure S8: GRHL2-KD circuit results for the investigated quantities. A)*** *Stacked histograms for GRHL2 in one replicate of the GRHL2-KD circuit at 10-fold down-expression (left panel) and the unperturbed circuit (right panel) with different phenotypes colored separately.* ***B)*** *Same as Fig S6A-C but for the GRHL2-KD circuit, with different levels of expression of the GRHL2 node.* ***C)*** *Same as Fig 4C-E but for the GRHL2-KD circuit, with different levels of expression of the GRHL2 node****. D)*** *Proportion of all states from monostable parameter sets belonging to different phenotypes in the GRHL2-KD circuit at different levels of expression of the GRHL2 node.* ***E,F)*** *Same as Fig S5 but for the GRHL2-KD circuit, with different levels of expression of the GRHL2 node. B-F: The values are averaged across replicates and the error bars represent the across-replicate standard deviations; for B-D, see* ***Table S8*** *in* ***Supplementary Section 2.5*** *for the statistical testing for differences).*

*
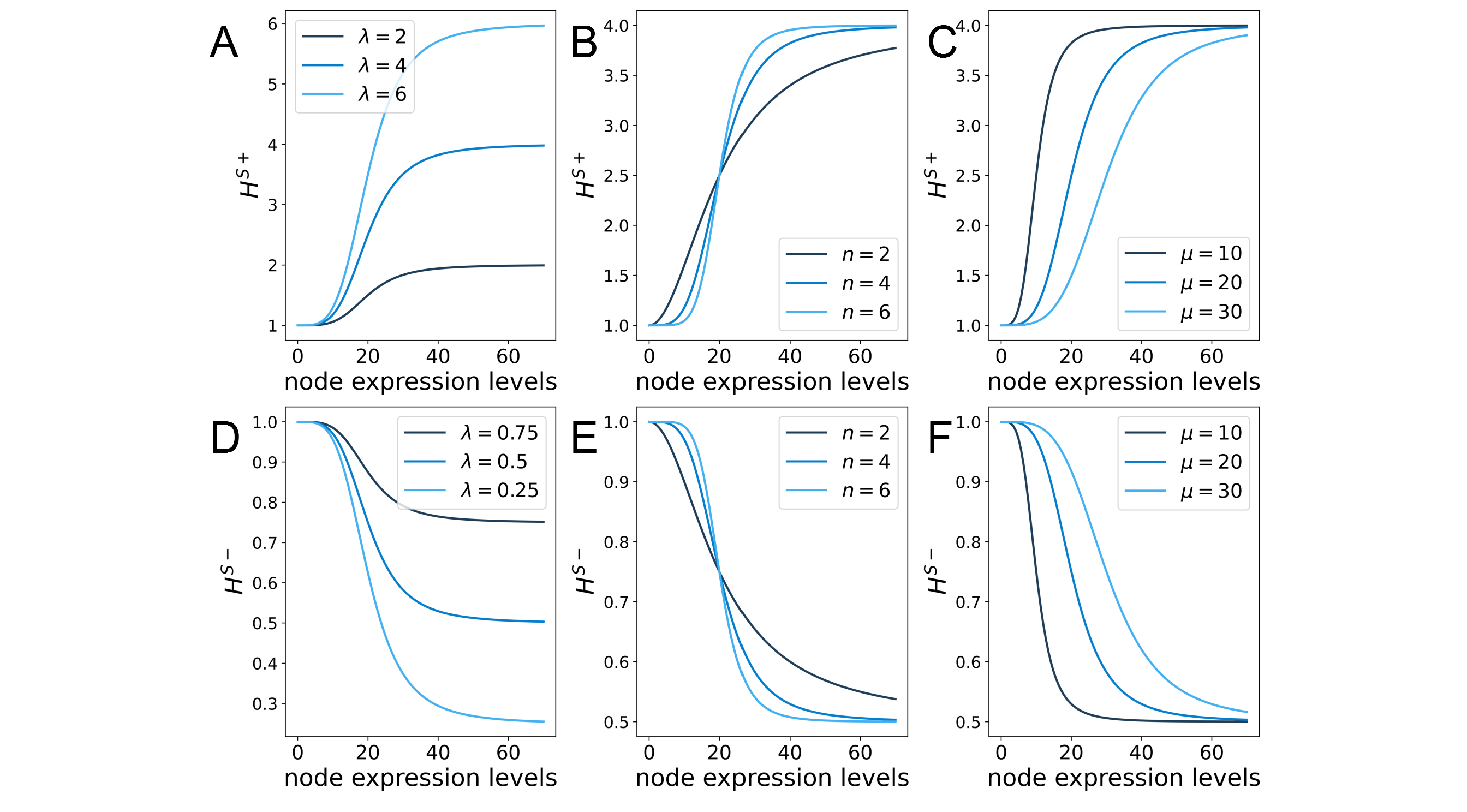
*

***Figure S9: Shifted Hill function across different parameters.*** ***(A-C)*** *Shifted Hill function for an activating link. A: μ=20, n=4 and varying λ, B: μ=20, varying n and λ=4, C: varying μ, n=4, and λ=4.* ***(D-F)*** *Shifted Hill function for an inhibiting link. D: μ=20, n=4, and varying λ, E: μ=20, varying n and λ=0.5, F: varying μ, n=4, and λ=0.5.*
